# Continuity of care for children with complex chronic health conditions: parents' perspectives

**DOI:** 10.1186/1472-6963-9-242

**Published:** 2009-12-21

**Authors:** Anton R Miller, Christopher J Condin, William H McKellin, Nicola Shaw, Anne F Klassen, Sam Sheps

**Affiliations:** 1 Department of Pediatrics, University of British Columbia, Vancouver, British Columbia, Canada; 2 Department of Anthropology, University of British Columbia, Vancouver, British Columbia, Canada; 3 Department of Family Medicine, University of Alberta, Edmonton, Alberta, Canada; 4 Department of Pediatrics, McMaster University, Hamilton, Ontario, Canada; 5 School of Population and Public Health, University of British Columbia, Vancouver, British Columbia, Canada

## Abstract

**Background:**

Continuity of care has been explored largely from academic and service provider perspectives, and in relation to adult patient/client groups. We interviewed parents of children with complex chronic health conditions to examine how their experiences and perceptions of continuity of care fit with these perspectives; and to identify the salient factors in the experience of, and factors contributing to, continuity in this population.

**Methods:**

Parents of 47 elementary school-aged children with spina bifida, Down syndrome, attention-deficit/hyperactivity disorder, Duchenne muscular dystrophy or cystic fibrosis participated in semi-structured interviews. Parents described and mapped the pattern of their interactions with service providers over time in all domains relevant to their child's health, well-being, and development (medical, rehabilitational, educational, and social supportive services), with particular attention paid to their perceptions of connectedness or coherency in these interactions. Verbatim transcripts were analyzed thematically using a framework approach to impose structure regarding parents' perspectives on continuity of care.

**Results:**

Existing academic concepts of relational, informational and management continuity were all discernable in parents' narratives. A thorough knowledge of the child on the part of service providers emerged as extremely important to parents; such knowledge was underpinned by continuity of personal relationships, principally, and also by written information. For this population, notions of continuity extend to the full range of service providers these children and families need to achieve optimal health status, and are not limited to physicians and nurses. Communication among providers was seen as integral to perceived continuity. Compartmentalization of services and information led to parents assuming a necessary, though at times, uncomfortable, coordinating role. Geographic factors, institutional structures and practices, provider attitudes, and, on occasion, parent preferences and judgments, were all found to create barriers to "seamless" management and provision of care continuity across providers, settings, and sectors.

**Conclusions:**

These findings add new perspectives to the understanding of continuity within chronically ill children's health care. They are relevant to contemporary initiatives to improve continuity of services to children with special health care needs, demonstrate the need for parental support of their important role in maintaining continuity, and suggest avenues for further research.

## Background

Improving health care for persons with chronic health conditions is a major goal of contemporary health service delivery systems [1]. Providing patients with a sense that the various elements of their health care services are connected over time and place, commonly referred to as continuity or coordination of care, is a key component of this goal [2]. Definitional, conceptual and measurement issues have hampered research and quality improvement initiatives involving continuity of care [3-5]. Nevertheless, several influential reviews of the past decade converge in affirming the primacy of personal relationships, information exchange, effective communications, seamlessness of services, and flexibility in responding to changing individual needs over time, in the construct of continuity of care [3,4,6].

Two widely cited reviews on continuity of care propose the following as key definitional elements: (a) it is an aspect of care experienced by persons receiving care, for services received over time; (b) it involves the patient's ("client's") experience of consistency, smoothness, and coordination in care; and (c) it relates to how patients/clients experience integration of services and coordination among providers [7,8]. The authors also delineated three main dimensions of continuity: relational continuity, which refers to an ongoing therapeutic relationship between a patient and one or more providers; informational continuity, defined as "the use of information from prior events and circumstances to make current care appropriate for the individual and his or her condition"; and management continuity, defined as the timely provision of services that complement each other within a shared management plan, delivered by a variety of providers. Management continuity emphasizes the use and consistent implementation of care plans, especially when patients cross organizational and service boundaries [7,8].

Much of the theoretical understanding of continuity of care, and empirical work on how it is experienced and perceived, is based on academic and provider perspectives in the fields of general practice [6,9], mental health [4] and nursing [10]. Less attention has been paid to the continuity of care experienced by parents of children with chronic health conditions since early studies showed that satisfaction with medical care is related to continuity of care, among parents of children with disabilities [11,12]. The gaps in understanding the perspective of parents of children with complex chronic health conditions are made more significant as a clearer picture of this population and its distinct service needs has emerged.

We are referring here to the 9 to 12% of all children and youth who may be affected by problems in more than one body system; who experience functional limitations as a result of medical condition; and who require health and related services beyond that required by children generally [13,14]. Affected children require an array of services that go beyond medical and nursing services, extending to rehabilitation, educational, social, and family support services [15,16]. Their parents are known to experience frustration as they try to weave through a complex and fragmented array of services that is difficult to manage [17,18]. In addition, planning for health and related services for children with complex chronic conditions must take into account important differences in the situation and needs of this population compared with adults [19,20]. Of particular relevance are developmental status and change during the childhood and teen years; the critical mediating role played by parents, and sometimes other family members, in seeking and implementing interventions that affect the child's health and well-being; and the role of the school environment as a context that shapes children's social development.

We addressed this existing gap in knowledge by undertaking a study of the experiences, perceptions and values of parents of children with complex chronic conditions as they relate to continuity of care. Using Reid and Haggerty's general framework [7,8] as a conceptual base, we aimed to examine two linked research questions: (1) to what extent can the constructs of relational, informational and management continuity be discerned in the narratives of parents seeking and receiving services for their children with complex chronic health conditions? (2) what aspects or elements of services are particularly salient to these parents' perception of care as continuous and connected?

## Methods

### Study design

This qualitative study used in-depth, semi-structured interviews to elicit parents' narratives about the care and services they received, and to map their interactions with formal and informal providers of health, developmental, educational, and social services. Parents' graphic representations of these interactions created opportunities for them to reflect upon their experiences and perceptions of continuity, coherency, and "connectedness" within service networks related to their child's care.

### Participants and recruitment

We used a purposive sampling strategy to recruit parents or primary caregivers of elementary school-aged children diagnosed with five chronic conditions: spina bifida, Down syndrome, attention-deficit/hyperactivity disorder (ADHD), Duchenne muscular dystrophy (DMD), and cystic fibrosis. Participants were contacted through specialized hospital clinics, physicians' offices, and patient support and advocacy organizations. These conditions were selected as representative of chronic conditions of childhood that have a significant and varied impact on child and family functioning and require a wide range of services [21]. In targeting this age group, we aimed to achieve a degree of comparability among participants in the length of time parents had had to reconcile themselves to the child's diagnosis, and to experience interactions with health, social, and educational services providers, while avoiding the issues inherent in negotiating transition to adult services. During recruitment through hospital and community recruitment partners, we emphasized our goal of achieving diversity within the parent participant group, while avoiding children with especially complex, multifactorial clinical profiles.

The participant group consisted of primary caregivers of 47 children, whose characteristics are summarized in Table 1. At this point in recruitment, subsequent interviews did not provide new information about the categories and themes under analysis. The participants were diverse in terms of geographic area of residence in the province of British Columbia (BC) and socio-economic backgrounds (informally assessed by observations of the state of the family home and parent reports of their occupation). Only one family did not use English as their first language. The vast majority of participating caregivers were parents, usually mothers, so we refer to all study participants as "parents." In one family however the child's guardians were grandparents.

**Table 1:** Characteristics of study participants

Interview participants (n = 47)	Mother only	26
	Father only	2
	Both parents (or grandparents)	19
Participants' health region of residence*	Interior	7
	Fraser	19
	Vancouver Coastal	8
	Vancouver Island	10
	Northern	3
Children's diagnosed health conditions	Cystic fibrosis	7
	Spina bifida	9
	Down syndrome	11
	Duchenne muscular dystrophy	9
	Attention-deficit/hyperactivity disorder	11
Age of children (years)	median (range)	9 (5-13)
Sex of children	Male:female	30:17

*British Columbia is divided into five regional health authorities

The study received approval from the University of BC Research Ethics Board, and, in accordance with University ethical guidelines, informed consent was obtained from all participants. All names used in this paper are pseudonyms.

### Contextual setting of the study

In BC, all children, including those with complex medical and developmental needs, have access to publicly-funded local and regional health care, as well as early intervention, rehabilitation, educational, and family supportive services. Psychology and other specialized therapy services are also available through the private sector. Physician and hospital services are provided under Canada's single-payer Medicare system. Specialized interdisciplinary clinics at the province's tertiary academic pediatric health centre offer longitudinal management and support to children and youth with cystic fibrosis, spina bifida and DMD.

### Interviews

**Figure 1:**
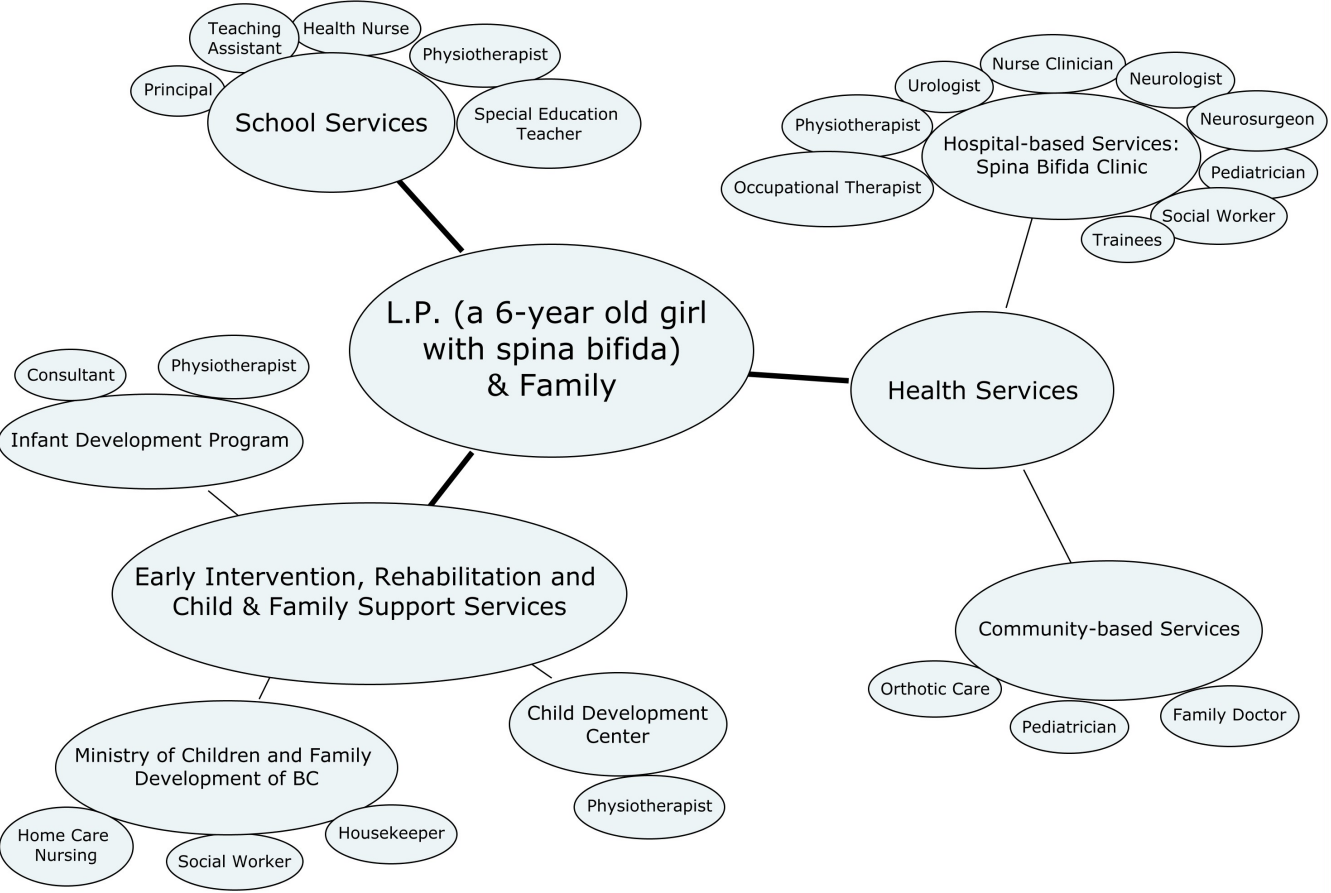
**Example of service network diagram**. *Notes: Interconnections among service providers are not shown*

Semi-structured, open-ended interviews were conducted by a trained member of the research team. Most families (44 of 47) were interviewed in their homes, while three were interviewed at the hospital at their request. Parents were encouraged to provide a spontaneous narrative about the various service providers with whom they and their child interacted over time, starting with their earliest contacts. Questions and probes were designed to provide an opportunity for parents to discuss how they perceived and experienced a number of aspects of their child's care (see Additional file 1: Interview Guide, Appendix). During the interview our goal was to elicit as complete a picture as possible of individuals' service providers, teams, networks, and agencies. To this end, parents were asked to draw a network diagram of all service providers. This diagram (see example, Figure 1), facilitated parents' explanations of how these services were connected or disconnected. All interviews were audiotaped and transcribed verbatim for analysis. The interviewer's field notes provided additional contextual information.

### Analysis

Transcribed interviews and field notes were imported into ATLAS.ti for data management and analysis of themes. Interview data underwent two major stages of coding and analysis. After 10 interviews were completed, three of the investigators representing the disciplines of pediatrics [ARM] and anthropology [CJC, WHM], respectively read the transcripts and met to develop a coding scheme, to identify themes for follow-up [22] in subsequent interviews, and to inform the data analysis phase of the study. A comprehensive coding scheme was then developed from the data to capture the broad range of parent experiences. Some codes were developed inductively given their repeated appearance in the interviews; others were derived deductively based on the Reid and Haggerty continuity model [7,8]. Because the dataset covered a large number of themes related to families' experiences of care more generally, we used a framework approach [23,24] to focus on themes and subthemes that were most relevant to continuity of care and continuity of services. This approach is well-suited to imposing structure on the data in a way that is most relevant to the aims of the study [23,24]. This more detailed coding was done on material that had initially been coded under broad headings of continuity--relational, informational, and management continuity--in the first round of analysis. All investigators contributed to an iterative process of theme identification and refinement.

## Results and Discussion

Six major overlapping themes emerged from parents' narratives of interacting with service providers over time. Within these narratives we discern the significance of relational, informational, and management continuity in the services that parents and their children had received over time. Their accounts further illustrate aspects or elements that were perceived as particularly salient to the continuity experience for these children and their parents.

### Theme 1. Relational and informational continuity and their significance

Relational and informational continuity, individually and in combination, were found to support at least three aspects of the care experience that parents value particularly highly: that service providers have as thorough knowledge of the child as possible; that service providers are able to relate to the child as effectively as possible; and that the child feels safe and supported in interactions with providers.

Knowledge of the child, according to parents, developed through relationships with a consistent set of service providers, both in and outside of medical settings. These personal relationships were augmented by written documentation and records. The parents of a child with cystic fibrosis described their confidence in a consistent group of clinical providers in this way: *"You need to see the regular faces, because they're the ones you feel at least know your child best," *the mother said. *"They know the history," *the father added, *"so you feel they have the whole story." *(07CF)

In a similar vein, the parent of a child with spina bifida described the importance of relational continuity with the person who provides the child's orthotic appliances: *"He [the orthotist] knows her. He knows her body, he knows how she moves. He knows how the bones are growing. He has seen her since she was born and followed her." *(01SB)

The father of a boy with ADHD and other behavioral difficulties spoke about relational continuity among the teaching and support staff at his son's school: *"They know what's going on. They know the history, and they're experienced with the history. They don't just go by hearsay or records or things on paper. They know interpersonally what makes this person tick." *(01ADHD)

We also noted interesting instances of relational continuity developing outside of the generally recognized networks of professional care. One parent, whose child has spina bifida recognized the school bus driver as a "core care provider" and contributor to their child and family's quality of life: *"That has made our life a lot easier, because [the bus driver] knows us so well, she'll go out of her way to do those little things that need to be done." *(01SB)

Parents alluded to perceived differences in the type, and perhaps quality, of the knowledge of the child acquired through interpersonal contact, compared with information in written reports. The parent of a child with spina bifida said, *"If someone else is just reading her file, then they don't really know her, right, and see how she's grown and progressed, or, you know, if she's getting better or worse. [People who know her] already know that sort of thing .... If someone else is coming in and just reading then [they] don't really know the whole facts." *(09SB)

However parents also acknowledged the role of written information, especially within environments in which there may be a lack of relational continuity among service providers. For example, the parent of a child with DMD commented, *"What has been nice is that there are such good notes and communication .... Somebody has taken enough time to write down everything and the next person has taken time to read it, so they're sort of on an even par with you when they come in." *(08DMD)

Consistent relationships with service providers over time are also perceived to benefit a child's sense of safety through contacts with providers who become familiar to them: "*It's nice when relationships do develop, you know. Kate knows the nurses [in the cystic fibrosis clinic] and she likes them, and ... she's not scared when she goes down there. Those faces are familiar to her, and if she is sick, it's not scary, it's not somebody she doesn't know." *(01CF)

Furthermore, service providers who know a child well are perceived by parents as better able to prevent and deal with challenging behaviors associated with chronic neurodevelopmental disorders and disabilities [25]. For example, referring to child and youth workers, the grandmother of a boy with ADHD and other disruptive behaviors said, *"When you've got a kid like Harry, or anybody for that matter, that's got any kind of problems, you need consistency, and you can't have somebody for six months and start all over." *(08ADHD)

Unfortunately, continuity is often not a feature of these kinds of relationships. As one parent pointed out: *"The doctor is constant, but you don't see the doctor all that often and the doctor doesn't know on a day-to-day basis what's going on. But the care providers that you have on a day-to-day basis, they change all the time." *(07DS)

For this population, the concept of continuity appears to be important across the complete network of services, not just for medical and nursing care. Relational and informational continuity interact to enable various kinds of providers to acquire a thorough, almost intimate, knowledge of the child that parents feel is needed for optimum care. Parents realize and accept that written information contributes to continuity of care, but knowledge garnered through consistency of personal contacts generates not only a more complex, contextualized appreciation of the child and family, but also a deeper understanding of the child's actual clinical characteristics. Repeated personal contact results in heightened sensitivity to physiological and functional changes that might be clinically relevant, but incorrectly attributed to individual variability or normal developmental effects. Relational continuity also allows providers to anticipate and deal with behavioral challenges more effectively, and for children to feel safe and comfortable in clinical settings.

### Theme 2. Continuity and communication

Communication is recognized as an important aspect of continuity in the literature [7,8,10], but there has been little description of how communication actually contributes to continuity from the point of view of patients/clients. In this study, parents identified communication as an integral feature of positive experiences of continuity of care, and described close and reciprocal links among communication, relationship-building, and continuity.

Several parents contrasted early intervention services with their subsequent experiences. The parent of a child with Down syndrome said that the only communication that happens in her son's elementary school is around his individualized educational plan (IEP)--*"Otherwise nobody talks to one another as to what's going on with Sam." *She contrasted this with her past experience at the child development center for pre-school children, where there was communication about the child and his needs *"going on outside of the meetings ... with everybody on an ongoing basis .... You basically get a sense that his needs are being taken care of and you don't have to worry about coordinating and making sure that you're not missing something." *(07DS)

The parents of a boy with ADHD lamented how communication had become attenuated during the school years: *"The teachers would communicate with each other. I find in the lower grades when you have a child with special needs, when they were changing classes, the teacher would say, you know, you're getting Frank next year .... That worked really good. When you get into middle school, they have three different teachers ... [and] the communication gets dropped." *(03ADHD)

One parent summed up the link between relational continuity and communication in response to a question about the nature of continuity itself: *"I believe that's what [continuity] is. It's a relationship. A relationship is formed on communication, you know, and that's all that's happening between a doctor and patient, for example ..." *(08ADHD)

Observations such as these extend our awareness of the broader types of interaction and communication that contribute to parents' experiences of continuity, and highlight how communication among providers appears to be a fundamental element in parents' experiencing services as connected or coordinated.

### Theme 3. Management continuity: seamlessness versus compartmentalization

Reid et al.'s [8] notion of management continuity encompasses an overall management plan or seamless connection among all providers in the patient/client's service network. Parents' narratives highlight key differences, however, between management continuity *within *a particular setting or service sector and continuity *across *settings and sectors. Parents often described high standards and even excellent management continuity provided by groups of service providers based in one location. However deficiencies in management continuity across settings, agencies, teams, or administrative service sectors were commonly identified. Their narratives frequently described compartmentalized services, evoking an image of multiple microcosms of service delivery. While each of these sectors could be functioning fairly well within itself, from the patient's point of view, each one is separated from the others within organizational "silos" [26].

Compartmentalization arose most frequently (but not exclusively) in parental accounts of poor linkage between the child's school program and medical services. Parents' narratives often evoked a sense of multiple management plans separated according to different areas of clinical-administrative responsibility, rather than an overarching plan for the child with special needs as a whole. For example, the parent of a child with Down syndrome and medical, learning, and behavioral challenges said, *"Luke has an IEP, and it's strictly for his education, but there's no medical [component] included in that. It's almost separated, like ... the behavior stuff is separate from the medical." *(05DS)

The parents of a boy with ADHD and hearing loss described their management plan as dealing with attention problems with use of medication, and mentioned a separate or different management plan for his education.

Even when information was transferred across agencies and sectors, this did not necessarily guarantee management continuity. For instance, while most of the children had files at school containing clinical information that parents perceived to be relevant to their child's educational programming, this resource was under-utilized. One parent asked a teacher if she had seen her child's file and was told, *"'Oh, I prefer not to look at it, because I only see the kids once a week, so I'd just rather deal with them as I see them.'" *(08ADHD)

In another case, the mother of a child with attention, learning, and mood problems described her interaction with a teacher about their child's plan: *"And she [the teacher] says, 'Oh, he had learning assistance last year?' and I said 'yes,' and she said, 'Well, I haven't read his file.' How can you help a kid when you have no idea what you're helping them with?" *(02ADHD)

Later in the same interview, the father pointed out that this happened every year, and the mother added, *"We have to go through the whole process again. So it's a good three or four months into school before we actually get him any kind of modification." *(02ADHD)

Compartmentalization was also described within a particular sector, evoking the image of silos within silos. The parent of a child with cystic fibrosis and permanent hearing loss noted that, *"Most of her stuff is [cystic fibrosis] stuff and then there's the hearing thing, but that's not a doctor thing, that's more of a rehabilitation, audiology, speech therapy, and that kind of thing. The two aren't really related, except when her delayed language might interfere with what a child her age can do in terms of their own care, because you can't really explain it to them." *(04CF)

Geography also emerged as a factor contributing to compartmentalization. Describing her perceptions of the connectedness of a specialized multidisciplinary hospital clinic and the physicians and other professionals in the child's home community, one parent of a child with cystic fibrosis said, pointing to different parts of the diagram of service providers, *"Basically, I see the clinic is here, and the doctors are here. They touch [pause], they touch [pause]. That's it. And it's not enough. They just kind of touch on the surface." *(07CF)

The mother of another child with cystic fibrosis, referring to the diagram of service providers, explained, *"There's a crease down the middle of the paper and I feel this half [the clinic professionals in Vancouver] deals really well with each other, and this half [the people who work in the child's home town] deals really well with each other." *The father added, *"You could almost call the crease a wall between the two." *(05CF)

As noted above, management continuity typically refers to connectedness among multiple service providers and patient/clients in the planning and providing of services. For parents of children with complex chronic health conditions, results from our study illustrate that the notion of management continuity could be extended to include the planning and information necessary to ensure health for the child in a more holistic sense. This point was illustrated by the parent of a child with spina bifida who had a motor disability, but who loved to ride a modified bike. She described problems she experienced getting the clinic to support the acquisition of a special bicycle: "*For her [the child, in the eyes of the clinicians]*, *it's just her medical care. Nothing to do with her emotional care, her quality of life." *(05SB)

Similarly, parents found it deeply frustrating not to be provided with sufficient information about services to address their range of needs. *"I think the individual health care workers do a good job," *the parent of a child with Down syndrome said. *"I mean, they do their work in their particular area and there's nobody really coordinating their work together. I see it as all the other stuff [social services and supports] that goes along with it that makes it all complicated and difficult." *(06DS)

The parent of a child with DMD said, *"Not only are you dealing with your child, you're dealing with the disease. You're dealing with the frustration of trying to get some kind of help, guidance, expert advice, and it's like two big jobs. You're finding out as much as you can about the disease and looking after your family, but you're also having to search, in all these different areas, for help." *(07DMD)

The ideal of management continuity for patients and families, across and within sectors and settings, is therefore challenged by entrenched structural, procedural, and attitudinal forces within and between organizations and providers.

### Theme 4. Parents working to ensure continuity

Parents frequently described the central, indispensable role they play in compensating for the systemic lack or breakdown of management continuity. They struggle to ensure and maintain continuity and coordination among a varied and disparate group of services. They provide informational continuity across geographically dispersed systems and between service sectors, acting at times as a conduit between different providers and institutions. They also serve as proxies for absent professional players and points of view at school, social or clinical services planning meetings, often physically carrying reports across professional settings.

The parent of a child with cystic fibrosis recalled the responsibility she felt for her daughter's care in a small community hospital: "*It was chaotic and frustrating, and nobody seemed to know what they were doing, and nobody was calling the specialized clinic at the Children's Hospital to find out what should be done. Then when we got down to the [provincial] Children's Hospital, they took it over from me, I didn't have to worry about it, it was all taken care of. I could just deal with Kate, and I didn't have to try and coordinate and make sure she was taking the right pills, they were doing this, and I didn't feel like I had to be in charge, like I did at the other hospital [where] I felt like they were going to do something that they shouldn't do, because they didn't know"*.(01CF)

Parents also reported having to play this role due to breakdowns in informational continuity between regular providers. The parent of a child with ADHD described follow-up visits to the family physician: *"Dr F [family physician] will look through his papers, and Dr P [pediatrician] hasn't sent him an update. So I'm updating him. And I don't really feel like that's my job to be doing that. It's between doctors to be doing that ... I don't have the wording that she does, being a pediatrician, to give Dr F. So I could omit stuff, or, it's not proper." *(11ADHD)

The father of a boy with ADHD said: *"I am the in-between guy. I am the guy that goes to the doctors and takes George and gets the meds, and well, the teachers told me this, and then they want to know what I see, and then the doctor comes up with the plan on what to do." *(01ADHD)

The parent of a child with Down syndrome explained the significance of the intermediary role parents play: "*It's very important for parents to know and to realize that they have to relay information, because other people may not have that information, right? Because even though the report may be sent, it may not be read. Or maybe the medication that was prescribed isn't in the report. Who knows, right? So, as the parent, you have to inform everybody about everything." *(10DS)

Some parents felt that service coordination was a critical role that parents should be comfortable with and skilled in, while others found it distressing. All, however, mentioned that this function was necessary to their child's optimum health and development; if parents did not do it, they did not know who would.

### Theme 5. Parents limiting continuity

Parents mitigate and compensate for the systemic breakdowns of relational, informational, and management continuity in the service system. At times, however, parents themselves create limits or impediments to continuity. While parents would sometimes express support for a completely seamless integration of ideas, information, and knowledge across settings, they also expressed a preference, or offered rationalizations, for some limits and control over the flow of information. Thus, the father of a boy with DMD said that he would like there to be one system, to which everyone could add information and have access; but later in the interview, the child's mother said, *"I don't want them to send [reports from the hospital] to the school. The school doesn't need to know until I think they need to know, and then I can tell them." *(05DMD)

Parents may also seek to regulate the flow of information between settings and providers whom they perceive as differing in their need for information about the child and/or family. These decisions are based on their own appraisals of which providers require more collaboration and communication. They also try to regulate their demands on professionals' time. The parent of a boy with Down syndrome and related ear and hearing problems talked about ensuring that the audiology reports go to the otolaryngologist, because he needs them, whereas the pediatrician probably doesn't: *"Are you just going to bog these people down with information and stuff that they don't need? Basically that's what we're doing, is making sure that the information is flowing that is necessary to flow." *(06DS)

Similarly, the grandparents of a child with ADHD and associated behavioral and learning difficulties felt that it probably wasn't necessary for the child's IEP to be sent to the doctors: *"There's enough people involved," *the grandfather said, *"... and they're busy enough anyway ..." *(12ADHD)

Research concerning the factors and circumstances that affect how parents (and patients) regulate informational continuity is limited. But a clearer understanding of parents' concerns (e.g. the selective distribution of information) is needed with the advent of models of service organization that emphasize greater integration between service providers, and the increasing availability of electronic health records and other technological tools to share information among providers.

### Theme 6. Systemic and organizational barriers to continuity

Key developmental transition points of childhood were often described by parents as events that cause changes in how services are organized. This often resulted in increased fragmentation of care, causing distress for parents. Children would "age out" of eligibility for certain programs, particularly rehabilitative and supportive services, leaving parents facing a lack of comparable services for older children, or a lack of coherence between the earlier and later programs. The parents of two different children with Down syndrome described the loss of early intervention services. The first explained that the provincial Infant Development Program (for 0-3-year-olds) provided information and coordinated the speech, physical, and occupational therapies. *"Once she hit three, though, all of that stuff fell apart, basically," *she said, *"Because then it was done through the local child development center, and [after that] it was all up to me ..." *(10DS)

The second parent described the transition to the school system: *"To me, it was like you were cut off from life. You turn six, that's it. You're gone. When they do it from zero to six, they coordinated. They stayed on top of it, they tell you what they need. As soon as they get into the school system ... I'm not even sure who coordinates it then." *(07DS)

Caregivers also described situations marked by lack of continuity stemming from overlapping and conflicting organizational mandates. The grandmother of a boy with ADHD and learning problems described the difficulty of dealing with the many "different areas" of social services: *"My biggest complaint about all of the resources is that they don't talk to each other. They truly don't talk to each other... And, every time, even if there's a worker who changes, you have to start right from the very, very beginning ..." *The child's grandfather added, *"The funding for the worker was coming from one place, and the funding for the daycare was coming from another place, and that was not coordinated well at all." *(12ADHD)

The policy environment is known to be an important factor in enabling or obstructing continuous and coordinated care at the system level [27,28]. Evidence about how these factors affect the day to day experience of children with complex chronic conditions and their parents adds immediacy to the need to address the policy problems.

## Conclusions

Theoretical constructs about the care of adult patients have dominated the discourse on continuity of care. Our analysis of the narratives and comments of parents of children with complex chronic health conditions therefore contributes to a relatively under-explored area of health services research. We identified six themes in parents' accounts that collectively represent salient aspects of parent's experiences and perceptions. Evidence from our interviews provides insight into the two key research questions we initially posed. First, we see that the key conceptual categories used to describe continuity of care in the adult and academic literature, are relevant for the analysis of the experiences of these parents. Though the terms relational, informational and management continuity are not typically part of parents' lexicons, these constructs were seen to be analytically useful in delineating aspects of parents' experiences of care. Second, our analysis reveals a broader and more complex conceptualization of care and continuity by the parents, than that framed by the literature. Parents' comments and observations illuminated dynamic interdependencies among the three types of continuity, as well as elements of experiencing care that are particularly, and in some cases, uniquely pertinent to perceptions of continuity and connectedness of care and services for this population.

Relational continuity was perceived as integral to the building of trusting, reassuring, and effective relationships with service providers, underpinned by a thorough knowledge of the child. Knowledge of the child, developed over time, emerged in this study as something parents saw as extremely important in their child's service providers. Parents appreciated the role of written information in creating bridges and continuity between providers, but pointed out differences in the kind or quality of knowledge and understanding of the child that is acquired through ongoing interpersonal contacts--an insight that has been previously noted by providers [6]. Informational continuity posed particular challenges for parents, as they stepped in to ensure this in the face of frequent systemic deficiencies and breakdowns. They perceive this as a necessary, if not always a welcome or comfortable, role for themselves.

Management continuity, was presented in parents' narratives as a contrast between idealized seamless and coordinated care on the one hand [29], and the realities of compartmentalized services, on the other. The compartmentalization that parents described seems more likely to occur between different kinds of teams working in different settings and service sectors. Conversely, parents perceived communication to be a core component of, and contributor to, continuity. A challenge, therefore, is to ensure that meaningful and effective communication occurs across geographic settings and administrative sectors, given time pressures, differences in "culture" and priorities, and the many other factors complicating the navigation of health care services [27].

Our findings suggest that, from the perspective of the patient/client/parent, continuity among multiple providers from different disciplines is possible, but that it seems to work best when there is collocation and functional integration of team players responsible for providing a functionally related set of services. These impressions are consistent with previous studies in which functional and financial integration [30], collocation of service providers [31], and interprofessional learning [32,33], all contribute to achieving more integrated models of health and social care, and improved communication and collaboration among diverse service providers.

Continuity of care has usually been approached as an aspect or dimension of health services referring to medical and nursing care. A critical implication of our study findings is that relational, informational, and management continuity should not be limited to physicians and nurses. We asked parents to describe their interactions with all the service providers needed for the child to achieve optimal health status, which, according to contemporary formulations, includes the child's subjective well-being and ability to perform daily activities and participate socially at home, in school, and in their communities [34]. Their narratives suggested the need for continuity to embrace this whole array of service providers. For this reason, the notion of "continuity of care" should perhaps be more accurately formulated as "continuity of services" for this population. Furthermore, connectedness between different kinds of intervention and supportive services and providers is experienced by parents not simply as a function of convenience or efficiency, but as an integral part of how the service system can help children and families cope. In this way, continuity is a key component of the "child-in-family" approach to chronic childhood illness and disability that is increasingly accepted as the standard of care for this population [15].

Our findings are a reminder of some major issues and barriers that face current efforts to promote more integrated, coordinated, and child-and-family-centered models of care. They emphasize that service delivery models remain for the most part organized around the priorities of organizations and institutions, rather than those of families. The parents in our study who mentioned, for example, the value to the family of relational continuity with the school bus driver, or of a specially modified bicycle for their daughter, illustrate the incongruity between the service system's traditional organizational notion of care, and the services and supports that parents may perceive as important. Similarly, administrative compartmentalization, funding polices that result in "aging out" of supportive services and programs, and constricted attitudes on the part of providers, all have a major impact on the ability of the parents to deal with the child and family's needs in a coherent and comprehensive way.

Certain findings from this study are relevant to the development of new models of care that emphasize coordinated services built around child and family needs [15,28,35], and care/service coordination strategies, such as provision of "key workers" [36,37]. Most parents were actively engaged in coordinating care for their children, and these important efforts warrant recognition and support. At the same time, parents brought different kinds of personal resources to this role. Some are better equipped and more comfortable with this role than others, and at times parents preferred just to be parents rather than semi-professional care coordinators. Some families may therefore need and welcome the support of a key worker more than other families, or at certain times more than others. Our study suggests that the responsibility for care coordination needs to be a negotiated interaction between service providers and family members and apportioned in a way that is most appropriate and supportive for each family.

We also observed how parents-as-clients may themselves play a critical role in channeling and managing the flow of information and communication in ways that sometimes may create and maintain separation and boundaries between service providers. Efforts to redesign service systems that emphasize coordination and continuity of care and services, within the broad objective of patient-centered (or in this study, family-centered) care, such as the "Medical Home" [35] or implementation of comprehensive electronic health records that links service providers across agencies and administrative silos, will need to be aware of and acknowledge parents' varying attitudes toward comprehensive and seamless models of informational and management continuity. Recent calls to vest ownership of the medical record in the hands of patients (or parents) as part of true patient-centeredness in health care [38] add further urgency to this issue.

The study had certain limitations. First, our recruitment strategy may have over-sampled families who were receiving hospital-based multidisciplinary care or connected with disease advocacy organizations. It is possible, therefore, that other points of view on continuity of services and its value were not as well represented. Also, in spite of success in recruiting families from diverse geographic and socio-economic backgrounds, we were able to enroll only one family that spoke English as a second language. There are known difficulties in reaching participants across linguistic and cultural barriers in research studies [39]. Nevertheless additional insight is needed on how such barriers may erode continuity of care for children among vulnerable or ethnically diverse populations.

Despite these potential limitations, the narratives of parents of children with complex chronic conditions and disabilities add an important and previously missing patient or parent-as-mediator voice and thus perspective to academic and clinical discourse in the field of continuity of care (or continuity of services). These narratives are relevant to short- and longer-term efforts to improve service delivery and organization for this as well as other related clinical populations, and also point to avenues of research need.

## Competing interests

There are no competing financial or commercial interests. ARM is involved in provincial and national initiatives to improve services for children and youth with complex health conditions and disabilities through improved service coordination and other strategies. NS is involved in research into continuity of care and continuity of information.

## Authors' contributions

AM conceived of the study, oversaw and coordinated all its phases, and took the lead in drafting the manuscript. All the authors participated in the design of the study, the analysis and interpretation of the data, and in writing and revising the manuscript. CJC was instrumental in data collection and organization.

## Pre-publication history

The pre-publication history for this paper can be accessed here:

http://www.biomedcentral.com/1472-6963/9/242/prepub

## Supplementary Material

Additional file 1 **Appendix**. Interview Guide: Overview of Interview Questions and Probes Click here for file
