# Supplementary material for: Continuity of care for children with complex chronic health conditions: parents' perspectives
Source: BMC Health Serv Res. 2009 Dec 21;9:242. doi: 10.1186/1472-6963-9-242 (PMC2805629; doi:10.1186/1472-6963-9-242)
Supplement: Additional file 1 — Appendix. Interview Guide: Overview of Interview Questions and Probes [file 1472-6963-9-242-S1.doc]

**Appendix**

**Interview Guide: Overview of Interview Questions and Probes**

| **Domain/Subject area** | **Objective(s)** | **Sample questions/probes used** |
| --- | --- | --- |
| Background on family & child | Basic biographical & demographic information | -Tell me about: age, education, occupation, number of family members, household occupants |
|  | Description and knowledge about child’s condition | -Tell me about your child’s condition. When was it diagnosed? What does it involve? How does it impact family life? Do you have a family history, or know other families with the same condition? |
| Interactions with service providers | Account of all SPs^*^ involved with child since diagnosis | -Let’s create a “master list” [network diagram] of all the people that you’ve seen. |
|  | Description of role played by each SP, type and quality of relationship with each SP | -Tell me how you came to see this SP? What role did they play? Tell me about your relationship with them; did it change over time? |
|  | Whether parent perceives a “core care provider” who oversees care (name, role, reason for identifying this person/organization) | -Is there one SP/organization in this diagram who is most involved with your child? Tell me about that person/organization. Why did you choose them? |
| Interactions between service providers | Perceptions of relationships between SPs | -How did/does SP X know SP Y? |
|  | Perceptions of information sharing between SPs | -How informed is/was SP Y about your child? How did you know this?  -Which SPs communicate with each other? How do they communicate? (e.g. by talking/writing etc.) |
|  | Perceptions of extent to which SPs share common understanding of condition/plan to address child’s needs | -Do your SPs share a plan to address your child’s needs? Do they recognize the same problems/symptoms?  -Do they share treatment plans with each other? How? |
| Parents’ overall evaluation of continuity | Evaluation of care and coherency in care | -Are there links in this network that are strong/weak? Why? |
|  | Meaning of “continuity of care” to parents | -There’s been a lot of talk lately about “continuity of care”. What does “continuity” mean to you? Why? |
| Other helpful people | Other actors important in providing support and/or care to child (family members, community groups, friends etc.) | -Who else has been important to you/your child? How have they helped you? |
| Concluding questions | Biggest challenges | -Are there some services / kinds of help that have been difficult to get? Which ones? Why? |
|  | Comparison of experience to others | -Do you think your experience has been typical of others? Why/why not? |
|  | Other topics that may not have been covered | -Is there anything that is important to you that I haven’t asked about? |

^*^SP=service provider

NOTE: Questions and probes are abbreviated to provide an overall sense of subject matter covered.
